# BioMeT and Algorithm Challenges: A Proposed Digital Standardized Evaluation Framework

**DOI:** 10.1109/JTEHM.2020.2996761

**Published:** 2020-05-28

**Authors:** Alan Godfrey, Jennifer C. Goldsack, Pamela Tenaerts, Andrea Coravos, Clara Aranda, Azid Hussain, Marcos E. Barreto, Fraser Young, Rodrigo vitório

**Affiliations:** 1 Computer and Information Sciences DepartmentNorthumbria University 5995 Newcastle upon TyneNE1 8STU.K.; 2 Digital Medicine Society (DiMe)BostonMA02114USA; 3 Clinical Trials Transformation Initiative (CTTI)DurhamNC27701USA; 4 Elektra LabsBostonMA02108USA; 5 Harvard-MIT Center for Regulatory ScienceBostonMA02115USA; 6 Groupe Speciale Mobile Association (GSMA)LondonEC4N 8AFU.K.; 7 Institute of Translational Medicine (ITM), University of Birmingham 1724 BirminghamB15 2THU.K.; 8 Computer Science DepartmentFederal University of Bahia (UFBA)Salvador40170-110Brazil; 9 School of MedicineOregon Health and Science University 6684 PortlandOR97239USA

**Keywords:** Digital health technologies, healthcare challenges, technology management, research and development, wearable sensors

## Abstract

Technology is advancing at an extraordinary rate. Continuous flows of novel data are being generated with the potential to revolutionize how we better identify, treat, manage, and prevent disease across therapeutic areas. However, lack of security of confidence in digital health technologies is hampering adoption, particularly for biometric monitoring technologies (BioMeTs) where frontline healthcare professionals are struggling to determine which BioMeTs are fit-for-purpose and in which context. Here, we discuss the challenges to adoption and offer pragmatic guidance regarding BioMeTs, cumulating in a proposed framework to advance their development and deployment in healthcare, health research, and health promotion. Furthermore, the framework proposes a process to establish an audit trail of BioMeTs (hardware and algorithms), to instill trust amongst multidisciplinary users.

## Graphical abstract

Proposal of a digital standardized BioMeT and algorithm evaluation framework (based on a software reference architecture approach) to overcome challenges in the field.

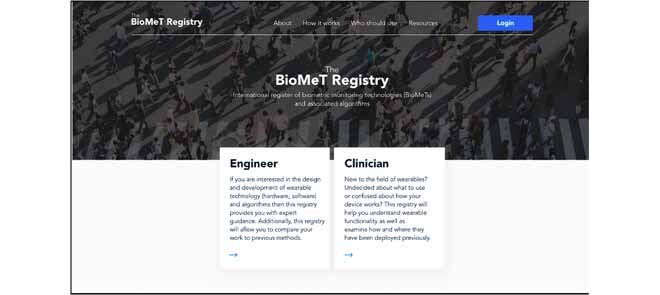

## I. Introduction

Advances in technologies are driving the development of low-cost, scalable digital solutions in modern medicine. Of course, not all technologies are created equal and a paucity of suitable guidance on how to make selections based on predefined clinical requirements is creating a bottleneck in the adoption of digital approaches in medicine; it is often difficult to know which product would be fit-for-purpose [1]. To date, there are a plethora of technologies available to healthcare professionals to monitor and track a range of biomedical data, outside of the clinic making them advantageous to study more habitual behavior’s and/or conditions due to a variety of different environmental contexts.

Biometric monitoring technologies (BioMeTs) are defined as connected digital medicine tools, processing data captured by mobile sensors using algorithms to generate measures of behavioral and/or physiological function [2]. BioMeTs capture person-specific data yielding objective digital measures that quantify human function to better monitor health and disease and enable digital phenotyping for enhancing clinical diagnosis [3]. Although there now appears to be a significant interest in the use of BioMeTs in research [4] they are not yet routinely used in clinical practice for many reasons such as usability, security and data privacy/governance issues as well as ongoing concerns about how to evaluate their quality (i.e. fit-for-purpose) and safety. The latter is the focus of this paper.

To enhance evaluation capabilities and promote the adoption of safe and effective digitally-collected measures, we: (i) discuss the rise of BioMeTs in digital medicine, as well as challenges and efforts to promote adoption; and (ii) propose a standardized BioMeT and algorithm evaluation framework (based on a software reference architecture approach) to overcome challenges in the field. The evaluation framework suggests procedures to advocate transparent approaches for BioMeT and algorithms.

## II. Challenge

### A. Biomet and Algorithm Challenges

BioMeTs and their corresponding algorithms are often created without expert guidance, disseminated and updated with a lack of transparency. Patients and care partners increasingly use mobile technologies, apps and social media to research information, identify treatment options and self-diagnose [5]. Consequently, health professionals are susceptible to increased pressures and demand based on technologies they may have limited knowledge of or experience using. There is a need to support knowledge exchange across a range of expertise and pragmatic tools to help guide clinical staff to make well-informed decisions, select appropriate BioMeTs (based on those that are fit-for-purpose [2]), thereby ensuring patient safety.

### B. Biomet and Algorithm Potential

BioMeTs can generate a variety of digital assessments spanning a broad range of diagnostic and prognostic measures, e.g. blood pressure readings to identify hypertension and reduced gait quality from accelerometry for fall prediction, respectively [6]. They offer high-resolution sensor-based data at scale and over time, augmenting traditional endpoints such as increased mortality risks [7]. The ability to deploy small, discrete BioMeTs that integrate with mobile platforms/smartphones may afford clinicians new insights. For instance, BioMeTs can be frequently could/can beyond the clinic, capturing objective free-living digital data not previously attainable with questionnaires or self-reported diaries, supplementing subjective opinions and experiences.

A profound example of how BioMeTs could better manage disease stems from studying motor control within Parkinson’s disease. Experts have described a variety of possible uses of inertial sensing BioMeTs in free-living/habitual environments [8]. They describe the benefits associated with quantifying walking/gait from high-resolution spatial and temporal data that could support a variety of applications including evaluating the efficacy of intervention, optimizing medication dosing, monitoring disease progression and cognitive decline [9]. The capture of such pragmatic, patient-specific information performed during habitual activities has driven an explosion of interest over the possibility of digital medicine to change how medications (and other interventions) are used, adjusted, and evaluated [10]. Here, we focus on inertial-based BioMeTs only as the inclusion of all types of devices would be beyond the scope of one paper.

### C. Inertial-Based Biomet and Algorithm Gaps

Many current clinical endpoints inadequately reflect patient burden compared to digital endpoints, the latter described as new armamentarium offering continuous rather than snapshot assessments [7]. However, the promise of BioMeTs is accompanied by a wave of algorithms and associated digital measures that are difficult to understand or evaluate in comparison to traditional outcomes. Some BioMeTs are clinically more intuitive than others, such as an instrumented approach to a timed-up-and-go test or total distance walked [11] and offer additional insights to traditional assessments under observation. Others, such as refined composite multiscale entropy measured during daily walking are not immediately translatable into current clinical practice despite showing value in assessing fall risk in older adults [12]. Yet, given the breadth of inertial-based BioMeTs and body attachment locations, there is little consensus on the exact algorithm and/or quantifiable measure that should be used for disease subtypes.

## III. Clinical Impact

In addition to the notable efforts and resources (section III) to offer insight to digital approaches in healthcare there are approaches to implement standardized reporting that are championed by scientific journals.^1^ However, all are not appropriate to evaluate digital-based BioMeTs or algorithms where multidisciplinary teams focus on different performance aspects of the technology. Here, we propose a framework based on a reference architecture which aims to be accessible to a variety of disciplines to ensure BioMeTs and their algorithms are adequately and transparently developed and understood. This involves a need to streamline BioMeT and algorithm development to aid more unified innovation and robust application in clinical cohorts. Given the prevalence of BioMeTs entering medical research, the general ease with which component algorithms can be created (often without scrutiny) and the wide professional interest in their use, bespoke guidance for a multidisciplinary audience is desperately needed. This could be achieved by creating a set of professionally tailored standardized guidelines and support mechanisms to ensure BioMeTs and algorithms are better understood and used appropriately within and across populations. Additionally, there is a need to track and trace (from engineering or computer science-based development through to clinical application) as well as improve the transparency of BioMeT and algorithms.^1^http://www.biomedcentral.com/getpublished/editorial-policies#standards+of+reporting

## IV. State-of-the-Art

Efforts to guide the creation, measurement, and evaluation of clinically meaningful outcomes are demonstrated by initiatives such as COMET (Core Outcome Measures in Effectiveness Trials): aiming to standardize core outcome sets across a wide range of health topics, representing the minimum that should be measured and reported in clinical trials for specific conditions [13].

Other generic sources/tools exist to help navigate the plethora of BioMeTs by providing general insights to functionality, e.g. Scripps Research Translational Institute open database of commercial products [14] and the National Health Service list of applications/apps to manage and improve health [15]. Yet, these sources lack information and expert guidance on verification/validation protocols for accurate and robust use. Other more appropriate and targeted information is found elsewhere. For example, the Clinical Trials Transformation Initiative (CTTI), co-founded by Duke University and the Food and Drug Administration (FDA), developed an open live database of feasibility studies to promote effective use of mobile technologies in clinical research to avoid duplication of existing research [16]. A non-exhaustive list of some current organizations and their resources to guide digital efforts in medicine are presented, grouped according to organization type, e.g. regulatory, non-profit, commercial, etc.:

• Clinical Trials Transformation Initiative (search Mobile Technologies): Develops and drives adoption of practices that will increase the quality and efficiency of clinical trials http://www.ctti-clinicaltrials.org
• WHO:
  • Digital Health Atlas: Global technology registry platform aiming to strengthen the value and impact of digital health investment https://digitalhealthatlas.org
  • Monitoring & Evaluation of Digital Health handbook: https://tinyurl.com/rrc2zny
• FDA (specifically: Medical Devices / Digital Health): Seeks to better protect and promote public health and provide continued regulatory clarity http://www.fda.gov
• National Health Service (NHS) Apps Library: Recommended Apps and tools to manage health and wellbeing http://www.nhs.uk/apps-library
• NICE (Evidence standards framework for digital health technologies): To make it easier for innovators and commissioners to understand what good levels of evidence for digital healthcare technologies look like http://www.nice.org.uk
• COMET: Development and application of agreed standardised sets of outcomes http://www.comet-initiative.org
• Equator Network: Seeks to improve the reliability and value of published health research literature http://www.equator-network.org
• Open mHealth: Global community of developers and health tech decision makers to help make sense of digital health data through an open interoperability standard http://www.openmhealth.org
• Open Wearables Initiative: Collaboration designed to promote the effective use of high-quality, sensor-generated measures of health in clinical research through the open sharing of algorithms and data sets. http://www.owear.org
• RANKED Health: Run by the Hacking Medicine Institute (HMi, a non-profit organization spun out of MIT’s Hacking Medicine program). This project is designed to review and rank healthcare focused applications http://www.rankedhealth.com
• Digital Therapeutics Alliance (DTA): Evidence-driven advancement of digital therapeutics with an industrial focus http://www.dtxalliance.org
• Digital Medicine Society (DiMe): Supports development of digital medicine through interdisciplinary collaboration, research, teaching, and the promotion of best practices http://dimesociety.org
• Elektra Labs: Advances remote patient phenotypic monitoring by enabling the safe and effective use of connected biosensors at home https://elektralabs.com
• Fair Sharing: Resource on data and metadata standards, inter-related to databases and data policies http://fairsharing.org
• Fitabase: Enable researchers to use the latest tools, devices, and apps to making it as easy as possible for researchers to measure, track, and engage participants http://www.fitabase.com
• NODE.Health: Evidence based digital medicine that brings together a network of societies, regulators, organizations and innovators https://nodehealth.org
• Personal Connected Health Alliance: Aims to advance patient/consumer-centered health, wellness and disease prevention http://www.pchalliance.org
• ReCODE: Set of policy recommendations for facilitating open access to research data http://www.dhi.ac.uk/recode/
• Scripps Research Translational Institute library: A resource for researchers and other stakeholders to learn about tools they might consider utilizing in health-related research or clinical practice https://digitalhealthlibrary.scripps.edu
• SMART Health IT: Previously proposed a universal API (application programming interface) to transform EHRs into platforms for substitutable iPhone-like apps https://smarthealthit.org
• Wellocracy: Information on new personal self-health technologies like activity trackers, wireless devices and mobile apps http://www.wellocracy.com

Although efforts by these organizations are useful, future work must link engineering and computer science-based studies and developments with clinical trials that adopt the same technology as well as provide information regarding the successful use of BioMeTs in drug approval and/or use in clinical practice. More specifically, structured evaluation frameworks are critical to ensure that ‘going digital’ will be a more trustworthy process, avoiding unnecessary barriers to technology adoption [2].

## V. Proposed Methods and Procedures

Co-authors hosted a 3-day workshop held in March 2019 (Newcastle, UK) involving a number of professionals from complementary disciplines including computing science, biomedical engineering, human-centered design, digital technologies, clinical and social sciences. Prior to the workshop, a scoping review of BioMeT literature was performed by attendees and used for points of framework synthesis discussion. When constructing the framework, a hybrid design approach was adopted [17] which emphasized the involvement of future end users expertise and experiences primarily for (i) the design and (ii) translational understanding and use of BioMeTs and algorithms in a clinical setting. Similar to other work in the field to evaluate measurement technologies [1], the approach adopted here was deemed most appropriate considering the multidisciplinary nature of BioMeT and algorithm research which requires input from a range of expertise in the field. Other collaborators from industry were later involved to ensure the framework proposed here aligned to current validation and verification initiatives [2].

Specifically, we set out to define a BioMeT and algorithm conceptual framework for a congruent reference architecture [18] to achieve the following aim:

I. To create a “tool” based on the design of congruent reference architectures to standardize and evaluate BioMeTs and their algorithms,

Once conceptual procedures were ratified, a prototype was developed with the following objectives:

i. Documentation of technical specifications;
ii. Ensure fit-for-purpose by creating transparency;
iii. Clarify verification and validation procedures and;
iv. Produce digital trails of BioMeTs and algorithms, so they may be tracked from development through to deployment for coherent and appropriate use.

Qualitative feedback on framework prototype design and functionality was acquired from healthcare professionals and computer scientists with experience of BioMeT use and algorithm development in older adult cohorts. The Faculty of Engineering and Environment, Northumbria University research ethics committee granted ethical approval (Ref: 9203). Participants gave written informed consent, agreeing to anonymized direct quotes being presented in this text.

### A. Workshop Scoping Analysis

Given the scoping review prior to the workshop it was found that current pragmatic resources to openly evaluate BioMeT and their algorithms are sparse. Although many are described in academic literature (and other online media), there are no standardized evaluation resources to direct how BioMeTs and algorithms are should be tested, verified, and validated according to expertly guided criteria with specific details of how each level of testing, verification and validation is performed. This includes but is not limited to robust data capturing protocols and statistical guidance. Additionally, no consideration is given to optimize the dissemination or evaluations of algorithms to the diverse, multidisciplinary audience utilizing them for pragmatic adoption. Current inconsistencies include algorithms reported in engineering-based pilot and feasibility studies for one cohort (e.g. younger adults) that are later used in a different cohort (e.g. older adults with stroke) where BioMeTs or algorithms may be unsuitable. Furthermore, discussion arising from the workshop highlighted three important implementation procedures to formulate the development of a robust conceptual framework: (1) clarity pertaining to BioMeT and algorithm descriptions, (2) audit trails (mapping) and (3) multidisciplinary approaches.

### B. Procedure 1: Biomet and Algorithm Descriptions

It was concluded that BioMeT and algorithm evaluation procedures proposed as part of the framework should be informed by extensive literature searches to ensure a rounded, well-informed and expert creation. This should be achieved by performing systematic reviews which target specific areas of BioMeT/algorithm development (e.g. inertial unit with accelerometers) and deployment (e.g. measuring gait). Systematically reviewing the literature within therapeutic areas should help categorize the range of BioMeTs and algorithms to quantify measures of interest for specific diseases. To categorize and prioritize findings from reviews, a Delphi method – an iterative process of expert review and feedback – should be used to identify the most appropriate details/content that should be known about a BioMeT and/or algorithm for inclusion on the framework, facilitating standardized testing protocols relating to expertly agreed verification, analytical validation and clinical validation procedures. It was argued that developing the framework in this way would ensure that details which are often lacking in peer reviewed literature – perhaps due to heterogeneity in reviewer experience and journal requirements/aims – are routinely identified and reported. This would ensure a well-informed, standardized evaluation and transparent reporting of details, yielding greater clarity on how and where BioMeTs should be used as well as limitations of their algorithms across various cohorts in different environmental conditions.

### C. Procedure 2: Digital Audit Trails and Mapping

It was deemed important that the framework provide mechanisms to register and track BioMeTs and algorithms through development and implementation. Currently, development of BioMeTs and their constituent algorithms cannot be robustly tracked across the literature. Often the only viable option is to thrall through references, locating and backtrack within the literature to find how and where BioMeTs and/or algorithms have been created and used. However, although some engineers are now adopting computer science-based approaches and placing code/algorithm online via open platforms (e.g. GitHub) this may still be a barrier for healthcare professionals who may struggle to engage with such a platform and/or be unsure about the implementation of code on BioMeT data. Specifically, for those unfamiliar with the field this is difficult and quite often pragmatic challenges exist such as access to relevant journal papers, which is a major limitation in developing economies. Moreover, slight variations in algorithms (e.g. thresholds) may often go unnoticed or unreported and there is often little detail or explanation as to why these occur. Implementing a framework that (i) assigns a unique identifier number to BioMeTs (its constituent sensors) and/or algorithms, and (ii) provide a mechanism to dynamically consider hardware or software updates would greatly help understanding and robust deployment. Such an approach would facilitate a central repository of BioMeTs and algorithms with an ability to track their development (version control), providing a digital audit trail useful for data scientists and clinicians alike.

Additionally, a mechanism to map the use of BioMeTs and algorithms was deemed necessary. Such functionality would list (map) where the technology has been used e.g. validation and later applied in cohorts. Such an approach would help clarify if appropriate validation procedures have been conducted for each cohort and allude to BioMeT/algorithm generalizability.

### D. Procedure 3: Multidisciplinary Functionality

The proposed framework aims to satisfy a range of research questions from diverse disciplines. In the first instance data scientists could use the framework to upload details of a BioMeT/algorithm based on a predefined set of questions (e.g. what make and model of accelerometer or what language was used to write the algorithm?). These questions would be developed through a separate Delphi processes for each field of research. When a new BioMeT or algorithm is entered into the framework, a new ID will be allocated. Updates to pre-existing BioMeTs and/or algorithms will be recorded as such. After details relating to protocols are entered, the algorithm will be uploaded and tested. If the algorithm is unique where no anonymized data exists in the framework, any data used by the engineer to develop the algorithm will be sought and required for upload also. Given said data may be limited to one/two test subjects, the framework’s administration team would collect independent data while also encouraging the research community to gather their own and contribute. Testing of algorithms and data should occur in a digital sandpit against the (read-only) reference data held by the framework to compare its accuracy against other similar algorithms and/or to its previous version (if applicable). The sandpit should provide some interactive functionality whereby the data scientist can manipulate his/her code to test new assumptions on the fly. Once testing is complete, the algorithm will be submitted to the framework administrators for final checking and later publishing to all framework users.

Clinical experts would experience more limited access to the framework. They should be able to search by therapeutic area or technology type and compare BioMeTs and algorithms. This will support selection of the most fit-for-purpose BioMeT.

### E. Development, Adherence and Impact

It was noted by workshop attendees that to populate and maintain the framework as a living resource, it should mimic existing efforts for registering and tracking research outputs (e.g. http://www.crd.york.ac.uk/PROSPERO). This will ensure the scientific community is aware of the framework as a useful “go-to-tool”.

## VI. Results

**FIGURE 1.**
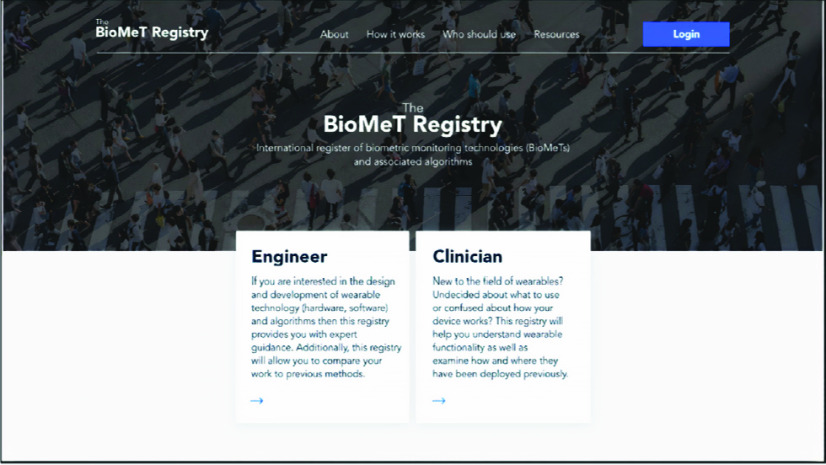
Framework title page. Tailored experience to (i) engineer (or data scientist) and (ii) clinician (healthcare professional), This dual engagement is a key feature to ensure widespread applicability to multidisciplinary users.

**FIGURE 2.**
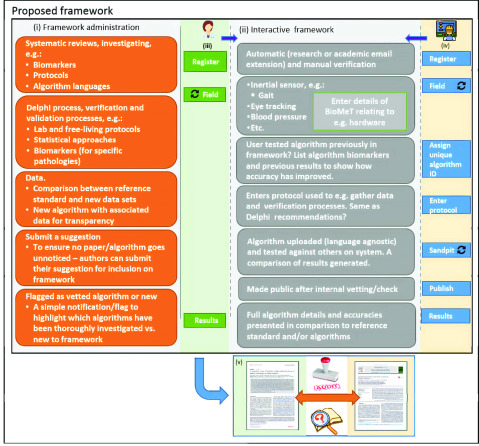
Proposed conceptual framework (left to right, top to bottom): (i) The framework should be informed by comprehensive systematic reviews from the literature in different therapeutic areas to inform dedicated BioMeT and algorithm development work in each pathology (Procedure 1). This work should extract e.g. digital measures and protocols to inform a Delphi process of expert agreement in each area to define appropriate evaluation standards, i.e. what measure and protocol should be quantified and used when creating new algorithms in a specific patient cohort. Algorithms should be accompanied by a selection of data used in their creation/design which could serve as a mechanism for other algorithms to be tested, useful for those with no resources to acquire participant data. (ii) The core functionality of the framework should align to multidisciplinary use with interactive features (Procedure 3). (iii) E.g. a healthcare professional registers and searches algorithms based on measurement of interest (e.g. gait) and views reports on how data were collected to verify and validate a gait algorithm. (iv) Engineers or data scientists should register and specify what pathology their BioMeT aligns to and what digital measurement it aims to quantify. New BioMeTs and algorithms should be assigned a unique identifier number (ID*) to tracking across the literature, providing an audit trail, Procedure 2. The framework could provide an interactive online sandpit to test algorithms, generating comparative results to other known algorithms in the field (Appendix A). (v) A digital audit trail would be useful to track BioMeTs and algorithms from areas of development (e.g. engineering) to application (e.g. health sciences), Procedure 2. * *ID could also be* $a$ *mechanism to track iterative improvements in any algorithm should it be updated across the literature*.

The proposed conceptual evaluation framework (design) details a digital interaction experience specifically tailored for differing professions who design/develop/create or use BioMeTs and/or their algorithms, i.e. engineers or data scientists and healthcare professionals, respectively (Fig. 1). Here, the framework is named the BioMeT Registry (Fig. 1 and 2) to highlight an objective, i.e. documentation of technical specifications.

Key to the successful creation and implementation of the framework is a robust administrative project management software layer (Fig 1-i) informed by systematic reviews and Delphi processes. Implementation of this layer will ensure robust iterative development, i.e. a procedure to broaden the framework to numerous areas of BioMeT development and application. For example, it was proposed by workshop delegates that the first iteration of the framework be created to harmonize and standardize inertial sensor-based BioMeTs and their quantification of gait/walking as that topic has shown particular pragmatic data capture in recent years for neurological cohorts (section II-B). Once initiated, the same methodologies (i.e. systematic review and Delphi process) could be used in other BioMeT areas of interest, e.g. electrocardiogram in coronary disease.

### A. Transparency and Tracking

**FIGURE 3.**
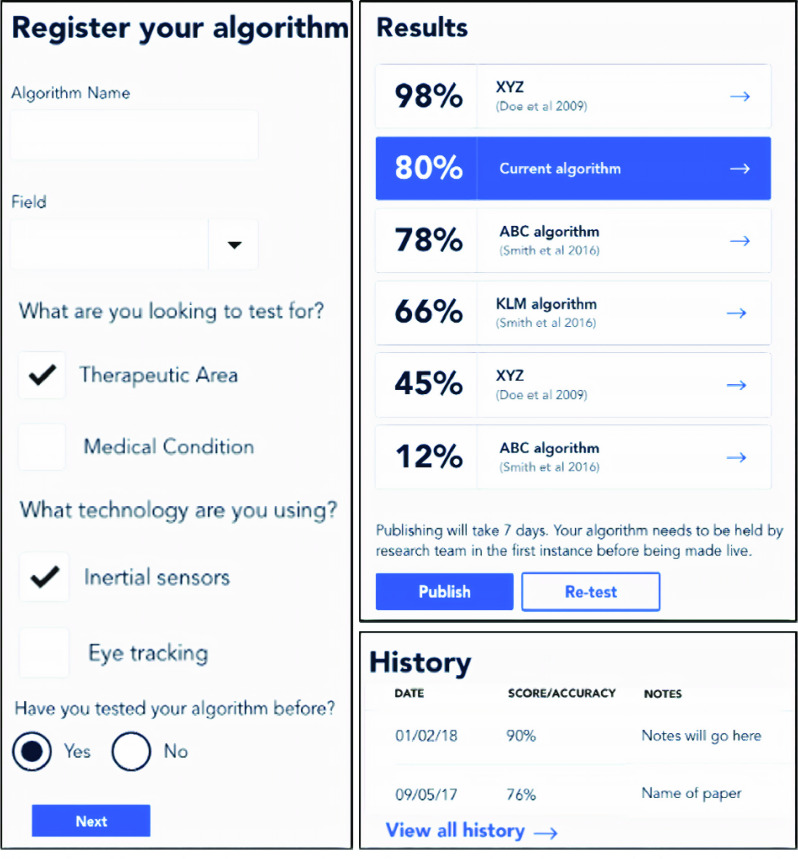
Key implementation framework characteristics include transparent registering of the algorithm (left) before examining accuracy results to other similar BioMeTs or algorithms within the same therapeutic area (top right). Any iterative BioMeT or algorithm improvements would also be recorded to see how technical adjustments aided increased accuracies (audit trail of BioMeT/algorithm, bottom right).

**FIGURE 4.**
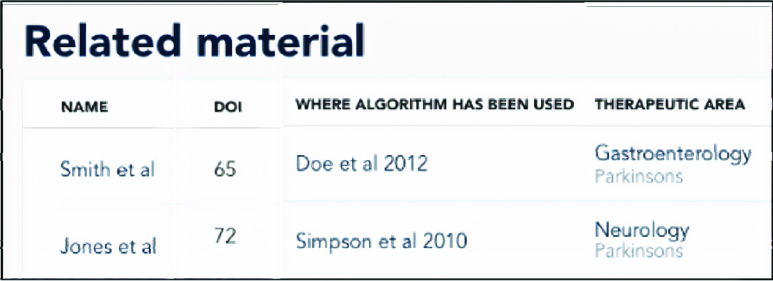
Mapping BioMeTs and algorithms used across the literature.

Procedures 1 to 3 are implemented to register, test, compare and track development and performance of BioMeTs and algorithms (Fig. 3). Such functionality should be observable from the engineering or clinical iterative perspective (Fig 1). Additionally, mapping and examining how and where BioMeTs and/or algorithms have been used across the literature is key, from engineering development to clinical application. All evaluation frameworks should support a digital trail to see if the technology has been developed and deployed in an appropriate manner (Fig. 3) and where it has been used (Fig. 4). For a full user flow experience please see appendices.

### B. Prototype Discussion

Qualitative feedback on framework prototype and general functionality for use to overcome challenges in the field was acquired from healthcare professionals (n = 2) and computer scientists (n = 2) with experience of BioMeT use and algorithm development in older adult cohorts. Users were presented with a description of the prototype, what it aimed to achieve and left to interact with it. A brief discussion captured user comments. In general, users indicated that the framework would “*provide* $a$ *very useful technique to examine wearables* [BioMeTs] *and to see where they have been used in one collective interface*”. One user liked the transparency of the framework but raised interesting insights, explaining that “*although the framework would be insightful,* $I$ *doubt* $a$ *typical clinician would understand how algorithms would function based on brief descriptions or code* [algorithm scripts] *displays*”. This raised an interesting point for discussion pertaining to the framework as an educational platform to offer the less technical user added insight to grasp the fundamentals of BioMeT/algorithm functionality. Upon further discussion, the user described a current online platform (http://www.codecademy.com) as one example that the framework/registry could draw inspiration from to implement a more focused translational learning experience. It was agreed that although useful, that would be beyond the scope of initial framework development and would require more tailored engagement from BioMeT and algorithm developers by adding more bespoke learning material upon registration.

Users praised version control aspects of the framework (Fig. 3, History) and spoke of similarities to GitHub. Certainly, the latter is becoming more popular with BioMeT and algorithm developers as the field moves towards more open methodologies [19]. However, simple version control logging representation presented here might “better appeal to those less familiar with code repositories”, who “would want simple and clear algorithm accuracy metrics and when they were achieved” (Fig. 3).

Lastly, implementation of the registry was discussed with notable challenges raised, primarily relating to framework administration (Fig. 2i) and scalability. Though it was described to users that the framework/registry should be created on inertial sensor BioMeT work for iterative aspects of physical functioning assessment (1. gait, 2. postural control etc), later progressing to other technologies for other physiological assessment, reservations were raised. “I think notable resources would be required for a single team to implement the registry at scale, given the complexity and time consuming nature of systematic reviews, including data extraction, and conducting Delphi exercises”. This raised a fundamental issue about implementing such a complex and interactive framework. Indeed, given its multifaceted nature, successful implementation may not be achievable unless significant financial resources were leveraged to supply personnel. Alternatively, it was discussed that the research community could look at this as an opportunity to harmonize the field and work collaboratively to share resources and responsibilities. Regardless, implementation of any such tool should follow methods/procedures proposed here to ensure rigorous approaches.

## VII. Future Direction and Actions

Attendees of a multidisciplinary workshop designed a novel framework that considers diverse expertise while implementing version control and digital auditing trails for inertial-based BioMeTs. The conceptual framework and resultant registry prototype suggests an approach to implementing harmonized and transparent approaches for BioMeT and algorithm development that aligns with existing initiatives in digital medicine promoting fit-for-purpose approaches. Implementing this proposed framework will be challenging, multifaceted and costly. Therefore, it is suggested that realization of such a clinically relevant and pragmatic engineering “tool” may only be feasible if considering a modular approach, i.e. separate groups taking leadership on different aspects of BioMeT work (i.e. different types of devices) in different therapeutic areas under the umbrella of this framework and its methodological procedures presented here. Moreover, this tool should be considered as one part of the process to ensure inertial (and other) BioMeT devices are used suitably as well as securely in healthcare applications. Although not the focus of this work, implementation of algorithms and analysis of patient data in applied healthcare applications should also consider best practice and expert direction for confidentiality and information security.

Currently, BioMeTs and their algorithms are being used without undergoing appropriate scrutiny and lacking expert guidance, generating heterogeneous and in some cases unvalidated and inaccurate digital measurements. In other cases, they are not being used at all as clinicians and technologists are unable to evaluate them. Our proposed framework would greatly help users from various backgrounds better understand and use BioMeTs and/or algorithms in the service of improved health, healthcare, and health research. The registry (Fig. 2) should be an open resource to implement evaluation standards in the field, ensuring digital measures are high quality, safe, effective, and fit-for-purpose.

## Appendix

See online supplementary material, Appendix A.
